# A brain-targeted, modified neurosin (kallikrein-6) reduces α-synuclein accumulation in a mouse model of multiple system atrophy

**DOI:** 10.1186/s13024-015-0043-6

**Published:** 2015-09-23

**Authors:** Brian Spencer, Elvira Valera, Edward Rockenstein, Margarita Trejo-Morales, Anthony Adame, Eliezer Masliah

**Affiliations:** Department of Neurosciences, University of California, San Diego, La Jolla, CA 92093 USA; Department of Pathology, University of California, San Diego, La Jolla, CA 92093 USA

## Abstract

**Background:**

Multiple system atrophy (MSA) is a progressive, neurodegenerative disease characterized by parkinsonism, resistance to dopamine therapy, ataxia, autonomic dysfunction, and pathological accumulation of α-synuclein (α-syn) in oligodendrocytes. Neurosin (kallikrein-6) is a serine protease capable of cleaving α-syn in the CNS, and we have previously shown that lentiviral (LV) vector delivery of neurosin into the brain of a mouse model of dementia with Lewy body/ Parkinson’s disease reduces the accumulation of α-syn and improves neuronal synaptic integrity.

**Results:**

In this study, we investigated the ability of a modified, systemically delivered neurosin to reduce the levels of α-syn in oligodendrocytes and reduce the cell-to-cell spread of α-syn to glial cells in a mouse model of MSA (MBP-α-syn). We engineered a viral vector that expresses a neurosin genetically modified for increased half-life (R80Q mutation) that also contains a brain-targeting sequence (apoB) for delivery into the CNS. Peripheral administration of the LV-neurosin-apoB to the MBP-α-syn tg model resulted in accumulation of neurosin-apoB in the CNS, reduced accumulation of α-syn in oligodendrocytes and astrocytes, improved myelin sheath formation in the corpus callosum and behavioral improvements.

**Conclusion:**

Thus, the modified, brain-targeted neurosin may warrant further investigation as potential therapy for MSA.

**Electronic supplementary material:**

The online version of this article (doi:10.1186/s13024-015-0043-6) contains supplementary material, which is available to authorized users.

## Introduction

The synucleinopathies are a heterogeneous group of neurodegenerative disorders that affect 5 million people worldwide and includes Parkinson’s disease (PD), dementia with Lewy bodies (DLB), neurodegeneration with brain iron accumulation, pure autonomic failure (PAF) and multiple system atrophy (MSA) (Reviewed in [1]). Multiple system atrophy is a rapidly progressive, neurological condition characterized by parkinsonism resistant to dopamine therapy, ataxia, autonomic dysfunction, and pathological accumulation of α-synuclein (α-syn) [2–4]. This disorder differs from other synucleinopathies in that α-syn accumulates not only within neurons and astrocytes, but also within oligodendrocytes in the form of glial cytoplasmic inclusions [5]. This intracellular accumulation of toxic α-syn species leads to degeneration of oligodendroglial cells, loss of trophic support to neurons and subsequent neurodegeneration.

The mechanisms through which α-syn leads to neurotoxicity are not completely clear, however recent evidence supports a role for oligomerization [6, 7]. Increasing evidence supports the notion that α-syn, which is primarily generated by neurons, can be toxic once released to the extracellular environment [6, 8, 9]. Extracellular aggregated α-syn can then propagate to other neurons and glial cells in a prion-like fashion [10, 11]. Although it had been previously suggested that the sole source of oligodendroglial α-syn was through endocytosis, a recent report showed α-syn mRNA in MSA oligodendrocytes suggesting that the origin of oligodendroglial α-syn might be both of endogenous nature and the result of propagation from neurons and/or other oligodendroglial cells [12]. Furthermore, propagation and accumulation of α-syn within astrocytes could lead to activation of these cells and subsequent neuroinflammation [13–15]. Therefore, the development of therapeutic interventions for MSA and related neurodegenerative disorders has been focused simultaneously on reducing α-syn accumulation, increasing α-syn clearance and preventing α-syn propagation.

Neurosin, (human kallikrein 6, KLK6, Zyme, Protease M), is a serine protease capable of cleaving α-syn [16–19]. This enzyme is found to be expressed throughout the body in many tissues [20] including the CNS in the choroid plexus and in oligodendrocytes and astroglial cells [21] of healthy individuals [20], as well as neurons and microglia of the hippocampus of Alzheimer’s disease patients [16, 22]. Down-regulation of neurosin is associated with accumulation of α-syn in patients with DLB/PD [23–25] as well in animal models of DLB/PD [25], whereas over-expression of neurosin in the brain via lentiviral (LV) vector reduces the accumulation of α-syn and improves neuronal synaptic integrity in an α-syn tg mouse model of DLB/PD [25].

Neurosin is expressed as a catalytically inactive pre-pro protein and is activated through autocatalytic proteolysis. Upon expression and secretion, auto-activation occurs first via cleavage of the pre-pro neurosin at Q19 followed by cleavage at K21, which produces the mature neurosin enzyme. The mature neurosin can then auto-proteolytically inactivate itself with cleavage at R80. This amino acid, when altered to Glutamine (Q), prevents the auto-inactivation generating a longer-acting enzyme [26]. *In vitro* proteolytic reactions with neurosin show that autocatalytic cleavage can begin as early as 10 min after incubation, and full cleavage to the inactive form occurs by 240 min incubation [26].

Since we have previously shown that stereotaxic injection of LV-neurosin into the hippocampus reduced the local accumulation of neuronal α-syn in a mouse model of DLB [25], for this study we sought to determine if gene therapy with this vector would also reduce the neurodegenerative process in a mouse model of MSA [27, 28]. However, given that the synucleinopathy in MSA is more disseminated throughout the CNS than PD, a more systemic approach is needed.

For this reason, we engineered the recombinant neurosin for systemic delivery with the R80Q mutation to allow for longer half-life in order that the active form of the enzyme would reach the BBB, transcytose the endothelial cells, and reach the site of α-syn accumulation before it gets degraded. In addition, we fused the 38 amino acid LDL-R binding domain from apoB; which we have previously shown to transport active enzymes from the blood to the brain [29–32]. In this instance we used a lentiviral vector to deliver the gene for NR-R80Q-apoB to the liver for systemic delivery. The liver acts as a depot organ to produce and secrete the active enzyme to the blood stream. Cells expressing large amounts of the LDL-R, including hepatocytes and Kupffer cells of the liver, will clear the majority of the recombinant protein from the blood. Binding to the LDL-R of these cells will result in clearance by ESCRT-III-mediated endocytosis leading to lysosomal degradation [29]. In contrast, at the BBB, the LDL-R will bind the recombinant protein and rather than target for degradation, will transcytose the protein to the neuronal side for release [33].

In this study, we show that the brain-targeted neurosin accumulated throughout the CNS in neurons, astrocytes and microglia, and resulted in reduced accumulation of α-syn in oligodendrocytes and astrocytes with possible clearance via microglia. It also ameliorated myelin degeneration, neuropathology and behavioral deficits. Thus, the modified, brain-targeted neurosin may warrant further investigation as potential therapy for MSA.

## Results

### Modifications to neurosin increase its stability without decreasing its ability to degrade α-syn

We have previously shown that neurosin, when expressed from a lentiviral vector is able to degrade α-syn and promote the survival of neuronal cells in culture when challenged with exogenous α-syn [25]. This vector was delivered by direct stereotaxic injection to an α-syn tg mouse model of DLB/PD. While this delivery route was efficient at delivering the enzyme to a localized area of the mouse brain, a more amenable route of delivery for the human brain might involve broad blood-based systemic delivery across the blood-brain barrier (BBB). For this purpose, we engineered neurosin with a novel BBB transport tag derived from the LDL-R binding domain of Apolipoprotein B, which we designated apoB [25, 29, 30, 34].

**Fig. 1:**
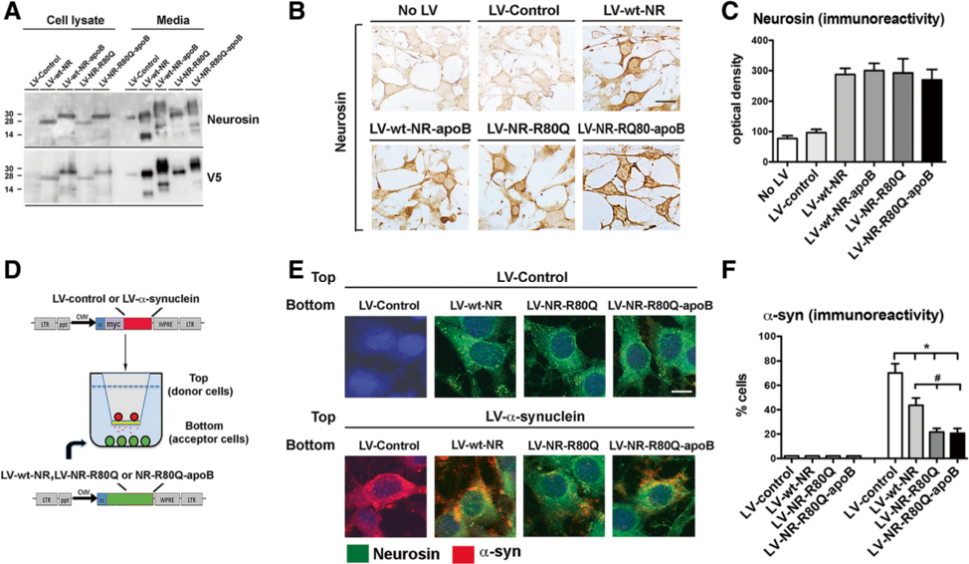
Lentivirus vectors over-expressing mutant neurosin reduce the accumulation and propagation of α-synuclein. Wild-type neurosin (LV-wt-NR), neurosin-apoB (LV-wt-NR-apoB), the point mutant neurosin (LV-NR-R80Q) and R80Q-apoB (LV-NR-R80Q-apoB) were cloned into the 3^rd^ generation lentivirus vector. **a** B103 neuronal cells were transduced with the vectors and examined by immunoblot for neurosin and the epitope tag V5. **b** B103 transduced cells were also immunostained for neurosin expression. **c** Compared to LV-Control, neuronal cells transduced with the neurosin constructs expressed higher levels of neurosin, levels were comparable between the wt and mutant neurosin constructs. **d** An *in vitro* neuronal co-culture system was devised to mimic the propagation of α-syn. B103 neuronal “Donor cells” infected with LV-α-syn or LV-Control (red) were plated in cell culture inserts containing a 0.4 μm membrane. B103 neuronal “Acceptor cells” infected with LV-NR-R80Q, LV-NR-R80Q-apoB or LV-Control were plated on coverslips. **e** Confocal microscopy and immunocytochemical analysis showing α-syn (red) from the Donor Cells was secreted and taken up by Acceptor cells that were double labeled with an antibody against neurosin (green). **f** Image analysis of double labeled neuronal cells, results are expressed as percent of acceptor cells containing α-syn immunoreactivity. * - indicates one way ANOVA with post hoc Dunnett’s, *p* < 0.05 compared to LV-Control. # - indicates one way ANOVA with post hoc Tukey-Krammer, *p* < 0.05 compared to LV-wt-NR. Scale bar = (B) 10 μm, (D) 5 μm

In addition to the apoB modification, the neurosin we previously delivered by stereotaxic injection was expressed in the same cells that α-syn was expressed, so long-lasting enzyme levels were not critically important to the design. Extracellular neurosin activity is regulated by an auto-proteolytic inactivation mechanism leading to a short half-life [26]. In order to increase the half-life of the recombinant protein to allow for systemic delivery and activity in the brain, we engineered a single point mutation at the site of auto-proteolytic cleavage (R80Q) [26]. This new protein was designated neurosin (NR)-R80Q-apoB. All neurosin vectors were tagged with the V5 epitope tag in order to differentiate recombinant expressed neurosin from endogenous neurosin expression. The media of cells infected with lentivirus expressing wild-type neurosin (wt-NR) and wild-type neurosin containing the apoB tag (wt-NR-apoB) contained several smaller sized bands associated with auto-proteolytic activity (Fig. 1a). In contrast, the NR-R80Q expressing vectors with or without the apoB tag showed significantly less auto-proteolysis product in the media of infected cells indicating an increased stability of the NR-R80Q (Fig. 1a). The NR-R80Q and NR-R80Q-apoB vectors were used to infect B103 neuronal cells, showing similar levels of immunoreactivity compared to the wt neurosin and wt neurosin-apoB (Fig. 1b, c). In a co-culture system designed to mimic propagation of α-syn from cells in the top (α-syn donors) to cells in the bottom (acceptors) (Fig. 1d), the acceptor cells that expressed NR-R80Q and NR-R80Q-apoB displayed significantly less accumulation of the α-syn secreted from the neurons in the upper chamber compared to cells expressing LV-Control (Fig. 1e, f). Together these results indicate: 1) NR-R80Q is able to reduce the propagation and accumulation of α-syn in acceptor cells, 2) addition of the apoB tag does not impair activity, and 3) the mutated version is more stable and active than the wt neurosin.

### Neurosin-R80Q-apoB transits to the CNS

**Fig. 2:**
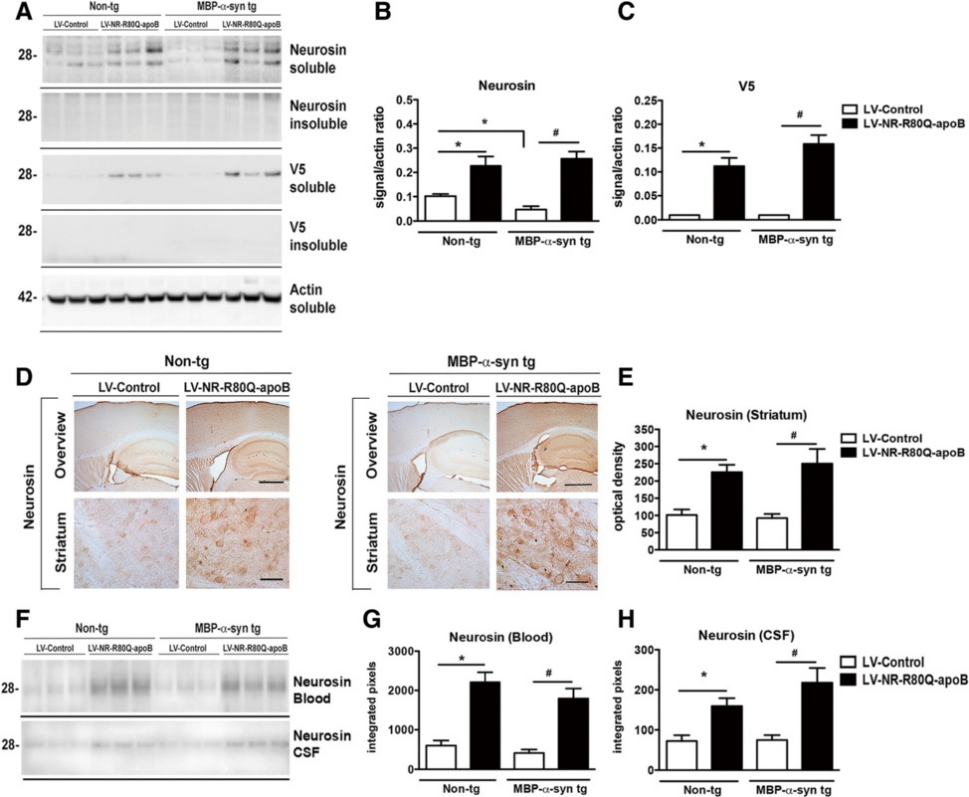
Neurosin distribution in the brain following systemic administration of LV-Neurosin-apoB vector into MBP-α-syn tg mice. Non-tg and MBP-α-syn tg mice received a single intra-peritoneal injection of LV-Control or LV-NR-R80Q-apoB (1 × 10^9^ TDU). Three months later mice were sacrificed, whole blood, CSF and brains were removed with half frozen for protein analysis and half fixed for tissue section immunohistochemistry. **a** Representative immunoblot from brain homogenates that were fractioned into soluble and insoluble fractions by ultracentrifugation and analyzed with antibodies for neurosin and the epitope tag, V5. Neurosin was detected as a double band at approximately 28 kDa. **b**, **c** Densitometry analysis of the levels of neurosin and V5 immunoreactivity plotted against the actin signal. **d** Hemibrains were serially sectioned on the longitudinal axis and immunostained with an antibody against neurosin. **e** Semiquantitative analysis of levels of neurosin immunostaining in the striatum expressed as optical density. **f** Representative immunoblot analysis of neurosin in plasma portion of the blood and in whole CSF. (**g**, **h**) Densitometry analysis of neurosin in the blood and CSF. *n* = 10 mice per group 9–10 m/o at the end of the treatment. * - indicates one way ANOVA with post hoc Dunnett’s, *p* < 0.05 compared to non-tg mice that received LV-Control. # - indicates one way ANOVA with post hoc Tukey-Krammer, *p* < 0.05 compared to MBP-α-syn tg mice that received LV-Control. Scale bars = Overview 200 μm, Striatum 40 μm

Non-tg and MBP-α-syn tg mice received a single intra-peritoneal injection of LV-Control or LV-NR-R80Q-apoB and 3 months later they were analyzed for the trafficking of the fusion protein neurosin-apoB to the CNS. Whole brain homogenates were fractionated into soluble and insoluble (membrane-bound) fractions and assayed by immunoblot for the presence of the recombinant neurosin. Neurosin was detected with both a neurosin-specific antibody and also with a V5 epitope antibody that is specific for the NR-R80Q-apoB protein. Mice treated with the LV-NR-R80Q-apoB displayed higher levels of neurosin in the soluble fraction compared to mice injected with the LV-Control (Fig. 2a, b). Comparable levels of neurosin were detected in non-tg and MBP-α-syn tg mice injected with LV-NR-R80Q-apoB (Fig. 2a, b). With the V5 antibody, neurosin was only detected in the soluble fraction of mice injected with LV-NR-R80Q-apoB but not in those injected with LV-Control, as expected (Fig. 2a, c).

Immunohistochemical analysis showed higher levels of immunoreactivity in the neocortex, hippocampus and the striatum in the non-tg and MBP-α-syn tg mice injected with LV-NR-R80Q-apoB compared to mice injected with LV-Control (Fig. 2d-e). In addition to detecting the NR-R80Q-apoB in the brain, following LV-NR-R80Q-apoB systemic injection, higher levels of neurosin immunoreactivity were detected in the blood and CSF of both non-tg and MBP-α-syn tg mice compared to mice injected with LV-Control (Fig. 2f-h). Specifically, a 2-fold increase in neurosin was detected in both the blood (Fig. 2g) and CSF (Fig. 2h) in animals that received the systemic LV-NR-R80Q-apoB injection.

**Fig. 3:**
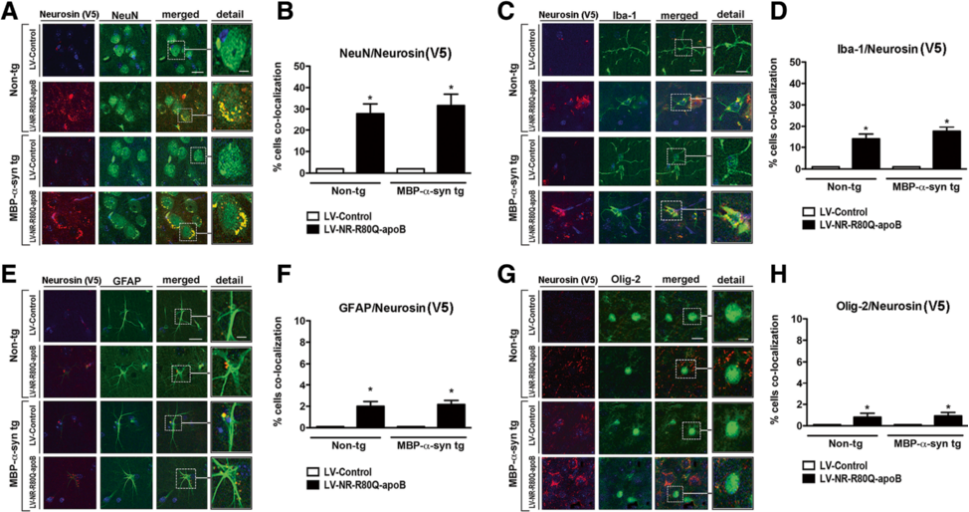
Cellular uptake of neurosin-apoB in the CNS following systemic delivery of the lentivector to MBP-a-syn tg mice. Non-tg and MBP-α-syn tg received a single i.p. injection with either LV-Control or LV-NR-R80Q-apoB. Three months later mice were sacrificed and brains were analyzed by double immunofluorescence and confocal microscopy for analysis of the co-localization of neurosin with cellular markers. Dotted box to the left depicts the image field zoomed represented under detail. **a** Double immunolabeling for V5 tag to identify neurosin (red) and the neuronal marker NeuN (green). Co-immunolabeling is represented by signal in yellow. **b** Computer aided image analysis of the % of NeuN cells displaying neurosin (V5). **c** Double immunolabeling for V5 tag to identify neurosin (red) and the microglial cell marker Iba-1 (green). **d** Computer aided image analysis of the % of microglial cells displaying neurosin (V5) immunostaining. **e** Double immunolabeling for V5 tag to identify neurosin (red) and the astrocytic marker GFAP (green). **f** Computer aided image analysis of the % of astroglial cells displaying neurosin (V5) immunostaining. **g** Double immunolabeling for V5 tag to identify neurosin (red) and the oligodendrocyte marker Olig-2 (green). **h** Computer aided image analysis of the % of oligodendroglial cells displaying neurosin (V5) immunostaining. All images are from the striatum. *n* = 10 mice per group 9–10 m/o at the end of the treatment. * - indicates one way ANOVA post hoc Dunnett’s *p* < 0.05 compared to non-tg mice that received LV-Control. Scale bar = 10 μm, detail = 20 μm

We have previously shown that apoB brain-targeted proteins are taken up by neurons and astrocytes [32]. To determine the cells to which the NR-R80Q-apoB localized, brain sections from mice treated with LV-Control and LV-NR-R80Q-apoB were double labeled with antibodies against the V5 tag in neurosin and the neuronal protein NeuN, the astrocyte marker GFAP, the microglial cell marker Iba-1, and the oligodendrocyte protein Olig-2. Compared to mice injected with the LV-Control, mice treated with LV-NR-R80Q-apoB displayed increased neurosin (V5) immunoreactivity in NeuN-positive neurons (Fig. 3a, b), and to a lesser extent increased immunoreactivity was also detected in microglia (Fig. 3c, d) and in astrocytes (Fig. 3e, f). However, in oligodendroglial cells only low levels of neurosin were detected (Fig. 3g, h). Since the NR-R80Q-apoB is targeted to bind the LDL-R, we performed co-localization immunostaining studies in the brains of the non-tg and MBP-α-syn tg mice to determine which cells expressed LDL-R (Additional file 1: Figure S1). LDL-R immunoreactivity was most abundant in neurons (NeuN), followed by astrocytes (GFAP) and microglial cells (Iba-1), which is consistent with the uptake of the NR-R80Q-apoB fusion protein (Additional file 1: Figure S1A, B). In contrast, oligodendrocytes (p25) showed the lowest levels of LDL-R immunostaining, suggesting that the low oligodendroglial uptake of the NR-R80Q-apoB protein (Fig. 3) is probably related to a low LDL-R expression (Additional file 1: Figure S1A, B).

### Neurosin-R80Q-apoB delivery to the CNS reduces α-syn accumulation in MBP-α-syn transgenic mouse model

**Fig. 4:**
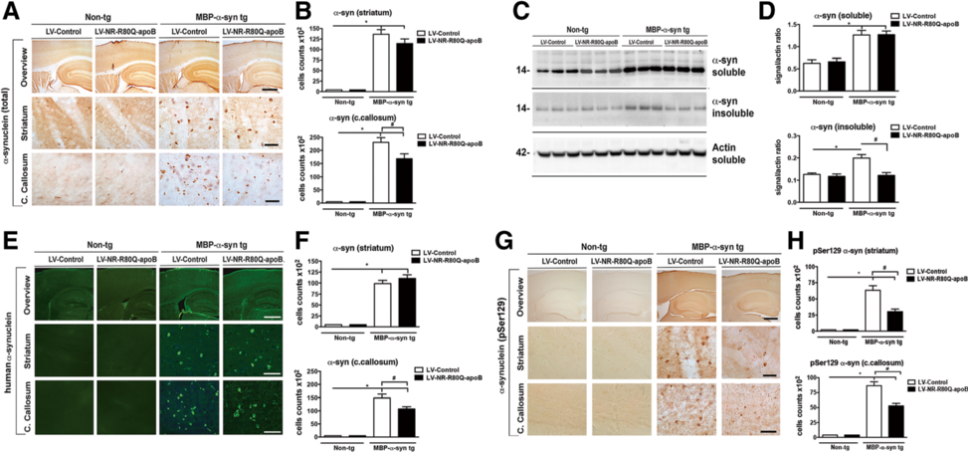
Delivery of LV-NR-R80Q-apoB reduces α-syn accumulation in MBP-α-syn tg mice. **a** Bright field microscopy analysis of serial longitudinal vibratome sections from the non-tg and MBP-α-syn tg mice immunostained with an antibody against total α-syn (syn-1) and analyzed for α-syn positive cells following treatment with LV-Control, or LV-NR-R80Q-apoB vector. **b** Computer aided image analysis for numbers of α-syn positive cells in the striatum and the corpus. **c** Samples that included the cortex, corpus callosum and striatum were fractioned by ultracentrifugation and analyzed by immunoblot analysis with an antibody against total α-syn (syn-1). **d** Densitometry analysis of the levels of α-syn immunoreactivity plotted against actin. **e** Laser scanning confocal microscopy of vibratome sections from the non-tg and MBP-α-syn tg immunostained with an antibody against human α-syn (syn211) and analyzed for α-syn positive cells following treatment with LV-Control, or LV-NR-R80Q-apoB vector. **f** Computer aided image analysis of the number of α-syn positive cells in the striatum and the corpus callosum. **g** Bright field microscopy analysis of serial longitudinal vibratome sections form the non-tg and MBP-α-syn tg immunostained with an antibody against phosphorylated α-syn (Ser129) and analyzed for α-syn positive cells following treatment with LV-Control, or LV-NR-R80Q-apoB vector. **h** Computer aided image analysis for numbers of phosphorylated α-syn positive cells in the striatum and the corpus callosum. Scale bars – top panels (low power, 40X) = 250 μm, bottom panel (high power, 400X) = 50 μm. * indicates statistical significance (*p* < 0.05, one way ANOVA, post hoc Dunnett’s) compared to non-tg mice LV-control. # indicates statistical significance (*p* < 0.05, one way ANOVA, post hoc Tukey-Kramer) compared to LV-Control treated MBP-α-syn tg mice

In the MBP-α-syn tg model, α-syn accumulates in oligodendrocytes in the neocortex, hippocampus, striatum, corpus callosum and brainstem [27]. To analyze the effects of the LV-Control or LV-NR-R80Q-apoB protein products on α-syn accumulation, brain sections were immunostained with an antibody against total α-syn (Fig. 4a, b). As expected, in the non-tg mice α-syn immunoreactivity was diffusely present in a granular like fashion in the neuropil. Similar levels of α-syn were detected in non-tg mice treated with LV-Control or LV-NR-R80Q-apoB, indicating that the viral vector-driven neurosin has no effects on the levels of endogenous α-syn. In the MBP-α-syn tg mouse, abundant α-syn immunoreactivity was detected in oligodendroglial cells in multiple brain regions including the striatum and corpus callosum (Fig. 4a, b). Compared to mice injected with the LV-Control, mice treated with the LV-NR-R80Q-apoB displayed a trend toward the decrease of α-syn in the striatum, and a significant reduction in the corpus callosum (Fig. 4a, b). To further confirm these findings by an independent method, brain homogenates were fractioned and analyzed by immunoblot with the same antibody against total α-syn. Consistent with the immunohistochemical analysis, levels of α-syn in the soluble fraction were comparable in the LV-Control and LV-NR-R80Q-apoB-treated non-tg mice (Fig. 4c, d). Levels of α-syn in the soluble and insoluble fractions were higher in the MBP-α-syn tg mice compared to non-tg, as expected. Treatment with LV-NR-R80Q-apoB resulted in decreased levels of insoluble α-syn in the MBP-α-syn tg mice (Fig. 4c, d). Additional confirmation of the results was performed with a different antibody specific for human α-syn (SYN211) (Fig. 4e, f). With this antibody, no endogenous murine α-syn is detected whereas abundant intracellular human α-syn accumulation is detected in the white matter tracts of the MBP-α-syn tg mice. Compared to LV-Control, treatment with LV-NR-R80Q-apoB reduced α-syn in the corpus callosum but not in the striatum (Fig. 4e, f). However, there was a significant reduction in phosphorylated α-syn (Ser129) in both the striatum and the corpus callosum (Fig. 4g, h).

Next, we analyzed if the reduced number of α-syn-containing oligodendrocytes in the animals treated with LV-NR-R80Q-apoB was related to proximity of these cells to cells containing the neurosin-apoB and/or to effects at the mRNA level. Double labeling/co-localization studies showed a reduction of α-syn in the MBP-α-syn tg mouse in the areas adjacent to where cells contained high levels of V5-tagged neurosin in mice that were treated with the LV-NR-R80Q-apoB vector (Additional file 2: Figure S2). In contrast, real-time PCR analysis of the MBP-α-syn tg mice treated with the LV-NR-R80Q-apoB vector showed no change in the transcription of α-syn mRNA indicating that the change in α-syn protein was due to degradation and not transcriptional alterations (Additional file 3: Figure S3). Consistent with this finding, double labeling and confocal microscopy studies with the oligodendroglial marker p25 and α-syn showed that the total number of oligodendrocytes were not significantly decreased in the striatum, however the levels of α-syn per oligodendroglial cell showed a trend to reduction between animals treated with LV-Control and LV- NR-R80Q-apoB, although this reduction was not statistically significant (Additional file 4: Figure S4A, B). Taken together, these results suggest that the intracellular level of α-syn within individual oligodendrocytes is not significantly affected by the treatment, and that the recombinant neurosin is primarily cleaving the α-syn that propagates from cell-to-cell.

### Neurosin-R80Q-apoB reduces the accumulation of α-syn in astrocytes and α-syn is cleared via microglia

**Fig. 5:**
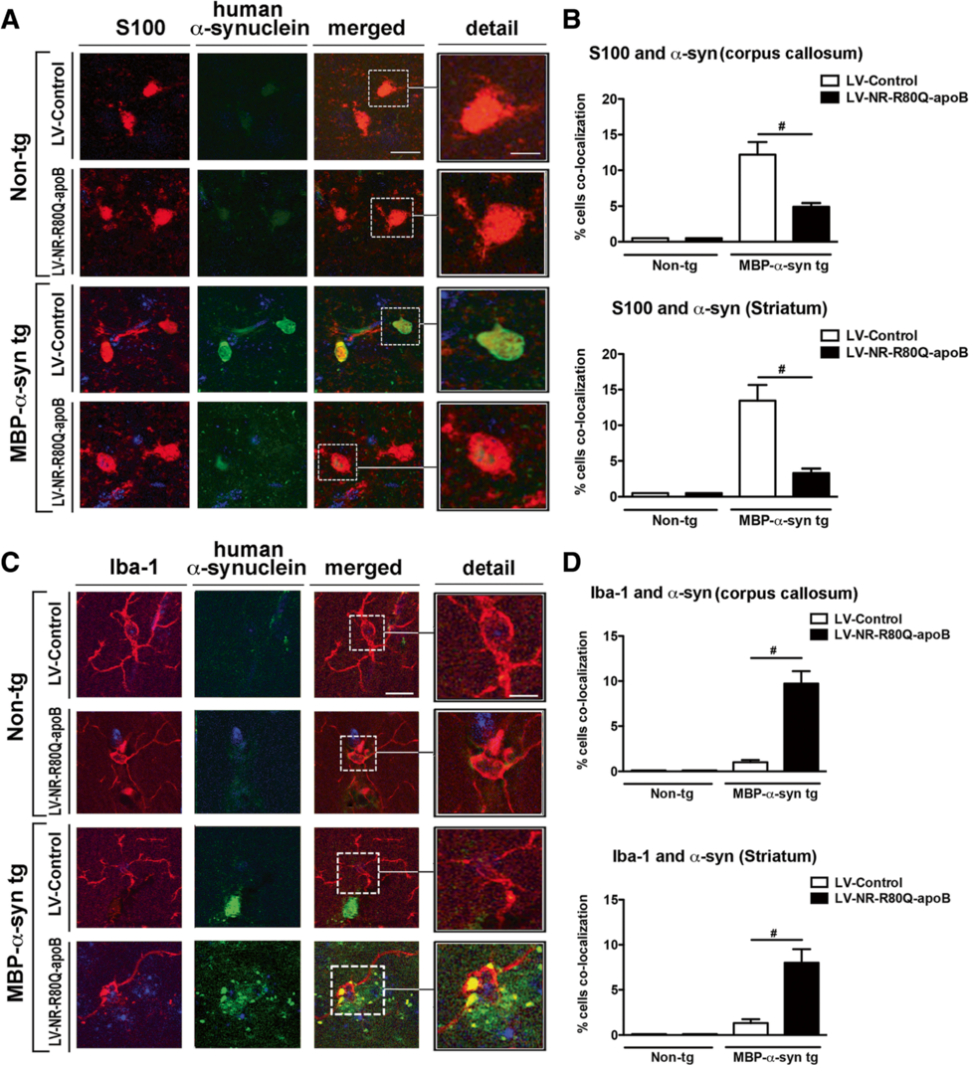
Delivery of LV-NR-R80Q-apoB reduces the propagation of α-syn to astrocytes and clearance via microglia in MBP-α-syn tg mice. Vibratome brain sections from the non-tg and MBP-α-syn tg that received i.p. injections of LV-Control or LV-NR-R80Q-apoB were double immunofluorescence labeled with antibodies against cellular markers and human α-syn and analyzed with the laser scanning confocal microscope with an optical image of 1 μm with fluorescent signals in co-registry. Dotted box to the left depicts the image field zoomed represented under detail. **a** Double immunolabeling for the astrocyte marker S100 (red) and human α-syn (green) with nuclei (DAPI, blue). Co-immunolabeling is represented by signal in yellow. **b** Computer aided image analysis of the % of S100 cells displaying α-syn immunofluorescence in the corpus callosum and striatum. **c** Double immunolabeling for the microglial marker Iba-1 (red) and human α-syn (green) with nuclei (DAPI, blue). Co-immunolabeling is represented by signal in yellow. **d** Computer aided image analysis of the % of Iba-1 cells displaying α-syn immunofluorescence in the corpus callosum and striatum. *n* = 10 mice per group 9–10 m/o at the end of the treatment. Scale bar = 10 μm, detail = 20 μm. # indicates statistical significance (*p* < 0.05, one way ANOVA, post hoc Tukey-Kramer) compared to LV-Control treated MBP-α-syn tg mice

We have previously shown that the astrogliosis and neuroinflammation observed in the MBP-α-syn-tg might be associated with propagation of α-syn from oligodendroglial cells to nearby astrocytes similar to propagation observed in MSA [13, 35]. To determine if treatment of the MBP-α-syn tg mouse with LV-NR-R80Q-apoB could affect the levels of α-syn in astrocytes, we examined the levels of α-syn in astroglial cells utilizing double labeling and confocal microscopy imaging approaches. Consistent with previous studies, in the LV-Control-treated MBP-α-syn tg mice there was co-localization of α-syn with the astroglial marker S100 in approximately 12 % of the cells (Fig. 5a, b). In contrast, treatment with the LV-NR-R80Q-apoB resulted in a marked decrease in co-localization of α-syn with the astrocyte marker, S100 (Fig. 5a, b). As expected, no α-syn was detected in the astrocytes of the non-tg mice. Since in previous studies we showed that extracellular α-syn can be cleared by microglial cells, next we performed double labeling and confocal microscopy imaging studies with the microglial marker Iba-1 and α-syn (Fig. 5c, d). In the LV-Control treated MBP-α-syn tg mice, only a few microglial cells displayed the presence of α-syn (Fig. 5c, d). In contrast, treatment with the LV-NR-R80Q-apoB resulted in a significant increase in co-localization between α-syn and the microglial marker Iba-1 (Fig. 5c, d). As expected, no α-syn was detected in the microglial cells of the non-tg mice. To confirm that microglia cells could take up neurosin-digested α-syn, we incubated oligomeric α-syn with lentivector expressed NR-R80Q-apoB and added this to BV2 microglia cells *in vitro*. Cells that received oligomeric α-syn alone accumulated α-syn around the periphery with little to no cellular uptake (Additional file 5: Figure S5). In contrast, BV2 microglia cells that received oligomeric α-syn previously digested with NR-R80Q-apoB showed endocytosis of α-syn and this protein was observed in punctate structures in the cytoplasm (Additional file 5: Figure S5). Taken together, these results suggest that LV-NR-R80Q-apoB reduced the accumulation of α-syn in astroglial cells and that microglial cells were further involved in the clearance of the extracellular α-syn.

### Neurosin-R80Q-apoB ameliorates neurodegenerative pathology in MBP-α-syn tg mice

**Fig. 6:**
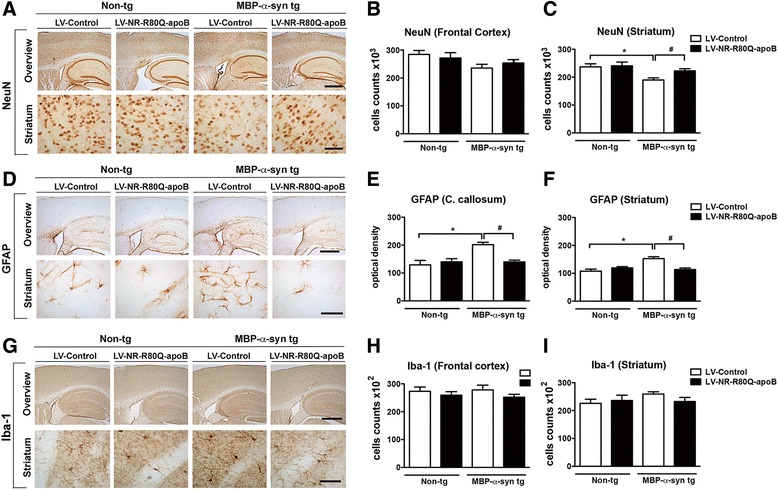
Delivery of LV-NR-R80Q-apoB ameliorates the neurodegenerative pathology in MBP-α-syn tg mice. Serial longitudinal vibratome sections were analyzed by bright field microscopy. Top row represents a low power overview (40X) of the section and panels in the bottom row show a higher magnification view (400X) of the striatum. **a** Immunohistochemical analysis with an antibody against the neuronal marker NeuN in brain sections of non-tg mice or MBP-α-syn tg mice injected with LV-Control, or LV-NR-R80Q-apoB vector. **b**, **c** Stereological analysis using the dissector method to estimate the total numbers of NeuN-positive cells in the frontal cortex and striatum. **d** Immunohistochemical analysis with an antibody against the astrocyte marker GFAP in brain sections of non-tg mice or MBP-α-syn tg mice injected with LV-Control, or LV-NR-R80Q-apoB vector. **e**, **f** Semiquantitative analysis of levels of GFAP immunostaining by optical density in the corpus callosum and striatum. **g** Immunohistochemical analysis with an antibody against the microglial marker Iba-1 in brain sections of non-tg mice or MBP-α-syn tg mice injected with LV-Control, or LV-NR-R80Q-apoB vector. **h**, **i** Stereological analysis using the dissector method to estimate the total numbers of Iba-1 positive cells in the frontal cortex and striatum. *n* = 10 mice per group 9–10 m/o at the end of the treatment. Scale bar = in the lower magnification panels 250 μm and in the higher magnification panels 50 μm. * indicates statistical significance (*p* < 0.05, one way ANOVA, post hoc Dunnett’s) compared to LV-control, non-tg mice. # indicates statistical significance (*p* < 0.05, one way ANOVA, post hoc Tukey-Kramer) compared to LV-Control treated MBP-α-syn tg mice

Previous studies have shown that widespread accumulation of α-syn in oligodendrocytes in the MBP-α-syn tg mouse model results in neurodegeneration in the cortex and striatum accompanied by neuroinflammation [27, 28]. For this purpose immunohistochemistry and image analysis was utilized to determine if delivery of the NR-R80Q-apoB would ameliorate the neuropathology and neuroinflammation. Consistent with previous results, compared to non-tg mice, MBP-α-syn tg mice treated with LV-Control displayed a significant reduction in NeuN cell counts in the striatum but only a trend in the frontal cortex; treatment with the LV-NR-R80Q-apoB ameliorated the loss of NeuN cells in the striatum to values similar to those observed in non-tg animals (Fig. 6a-c) treated with the LV-Control or LV-NR-R80Q-apoB vectors. In agreement with these results, compared to non-tg mice, the MBP-α-syn tg mice treated with LV-Control showed significant astrogliosis (GFAP) in the corpus callosum and striatum. Treatment with the LV-NR-R80Q-apoB vector significantly reduced astrogliosis in the corpus callosum and striatum to values similar to those observed in non-tg animals (Fig. 6d-f). At the age these animals were analyzed microgliosis was also not altered in either the frontal cortex or striatum of MBP-α-syn tg mice when compared to non-tg controls. Treatment with the LV-NR-R80Q-apoB vector did not appear to alter microgliosis in either treated animal group indicating that the treatment itself does not promote an adverse inflammatory response (Fig. 6g-i, Additional file 6: Figure S6).

### Neurosin-R80Q-apoB ameliorated myelination deficits in the MBP-α-syn tg mice

**Fig. 7:**
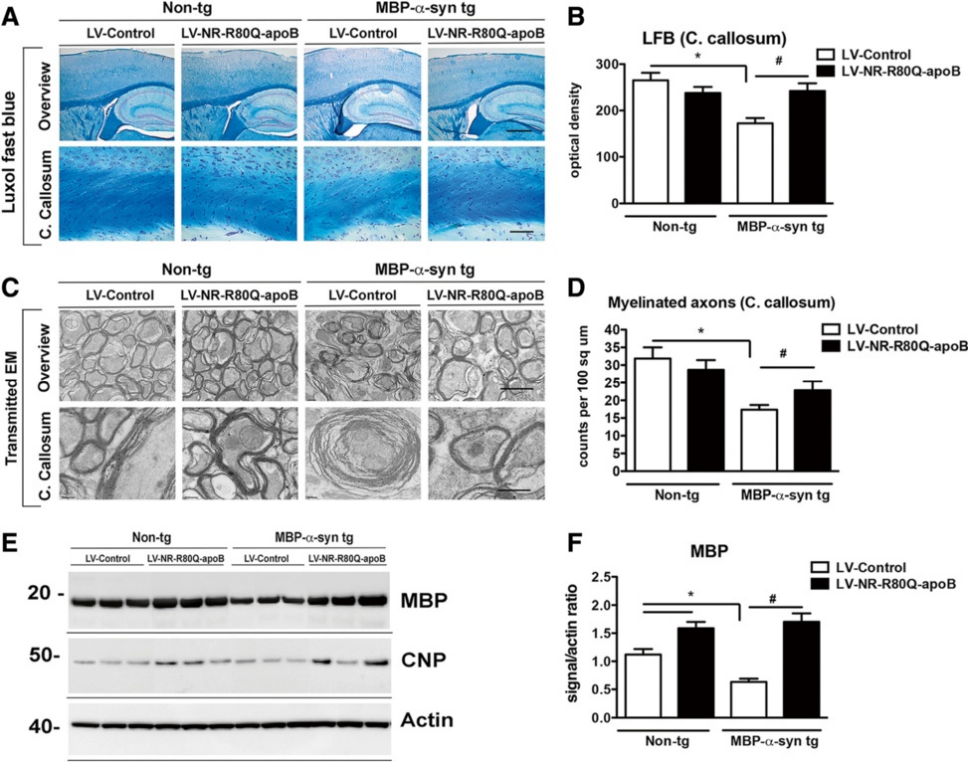
Delivery of LV-NR-R80Q-apoB reduces demyelination in MBP-α-syn tg mice. **a** Vibratome brain sections were stained with luxol fast blue (LFB) and analyzed by bright field microscopy. The panel in the top is a lower magnification view (40X) showing myelin in blue in white matter tracks. The bottom panels are higher magnification (400X) of the corpus callosum of non-tg mice or MBP-α-syn tg mice treated with LV-Control or LV-NR-R80Q-apoB. Scale bars = Overview 250 μm, corpus callosum 50 μm. **b** Semiquantitative analysis of LFB staining by optical density in the corpus callosum. **c** Transmission electron microscopy (TEM) images of myelin sheaths in the corpus callosum of non-tg mice or MBP-α-syn tg mice treated with LV-Control or LV-NR-R80Q-apoB. Representative micrographs taken with TEM are shown at low magnification (5,000x) and high magnification (25,000x). Scale bars = Overview 2.5 μm, Corpus callosum 500 nm. **d** Computer aided image analysis of the number of myelinated axons in corpus callosum. **e** Tissue blocks containing the corpus callosum and striatum were fractioned by ultracentrifugation and analyzed by immunoblot with antibodies against MBP and CNP. (**f**) Densitometric analysis of the MBP and CNP signal normalized to the actin signal. * indicates statistical significance (*p* < 0.05, one way ANOVA, post hoc Dunnett’s) compared to LV-Control treated Non-tg mice. *n* = 10 mice per group 9–10 m/o at the end of the treatment. # indicates statistical significance (*p* < 0.05, one way ANOVA, post hoc Tukey-Kramer) compared to LV-Control treated MBP-α-syn tg mice

The accumulation of α-syn in oligodendroglial cells is associated with demyelination in patients with MSA [36]. To examine the effect of treatment with LV-NR-R80Q-apoB on myelination in the MBP-α-syn tg mice, sections were stained with luxol fast blue (LFB) and myelination was analyzed in the neocortex, corpus callosum (Fig. 7a, b) and striatum. Consistent with previous studies in these mice [27, 28], the MBP-α-syn tg mice display reduced levels of staining with LFB in the corpus callosum compared to non-tg controls (Fig. 7a), indicative of myelin disruption in these mice. Treatment of the MBP-α-syn tg mice with LV-NR-R80Q-apoB was associated with increased levels of LFB staining in both neocortex and striatum (Fig. 7a, b) to values similar to non-tg controls. These results were further confirmed by analysis of the myelin sheaths by electron microscopy in corpus callosum (Fig. 7c). In the non-tg mice, the myelin sheath can be observed as a highly organized multilaminar structure (Fig. 7c). In the LV-Control MBP-α-syn tg mice, myelinated axons were reduced, the myelin sheath had fewer layers and was substantially more disorganized compared to the non-tg mice (Fig. 7c). In the MBP-α-syn tg mice treated with LV-NR-R80Q-apoB, the number of myelinated axons as well as the number of myelin layers per axon were preserved and their levels were comparable to non-tg controls (Fig. 7c, d).

Immunoblot analysis of myelin basic protein (MBP) from dissected brain homogenates revealed a significant reduction in total MBP protein in the LV-Control treated MBP-α-syn tg mice compared to non-tg mice (Fig. 7e, f). Treatment with LV-NR-R80Q-apoB increased levels of MBP protein to levels greater than those in non-tg mice (Fig. 7e, f). The MBP protein levels increased at the post-transcriptional level, as real-time PCR analysis of the LV-NR-R80Q-apoB treated MBP-α-syn tg mice showed no change in transcription levels of MBP mRNA (Additional file 2: Figure S2B). Furthermore, CNP protein expression is associated with increased oligodendrocytes process extension and myelination [37]. Compared to the LV-Control-injected MBP-α-syn tg mice, CNP protein levels were increased in MBP-α-syn tg mice treated with LV-NR-R80Q-apoB indicating that the presence of elevated neurosin improved the oligodendroglial process extension and branching in the MBP mouse model (Fig. 7e).

### Neurosin-R80Q-apoB reduces behavioral deficits in MBP-α-syn tg mice

**Fig. 8:**
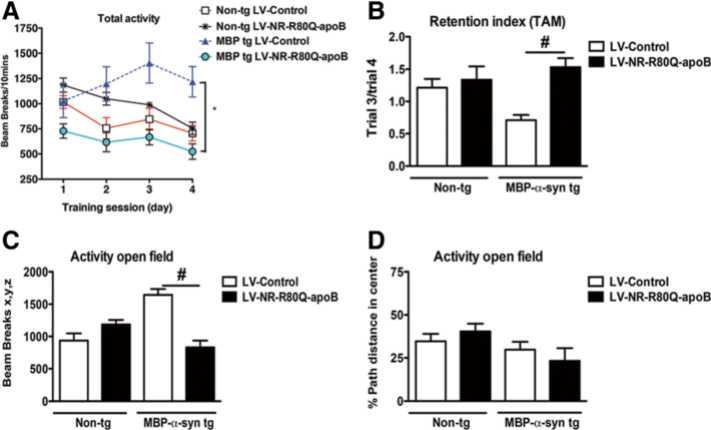
Treatment with LV-NR-R80Q-apoB improves the behavioral deficits in MBP-α-syn tg mice. Non-tg mice or MBP-α-syn tg mice treated with LV-Control or LV-NR-R80Q-apoB were tested in the open field test for 4 successive days or for a fixed period to ascertain the functional effects of LV-NR-R80Q-apoB treatment. **a** Total activity after consecutive days was performed, this is a test of habituation to a novel environment and of memory acquisition. **b** Retention score analysis representing the ratio between trials 3 and 4. **c** Total spontaneous activity after 10 min in the open field test. **d** Total activity in the center of the cage, no differences were noted among the 4 groups. *n* = 10 mice per group 9–10 m/o at the end of the treatment. Scale bar = 10 μm. * indicates statistical significance (*p* < 0.05, one way ANOVA, poshoc Dunnett’s) compared to non-tg mice. # indicates statistical significance (*p* < 0.05, one way ANOVA, post hoc Tukey-Kramer) compared to LV-Control treated MBP-α-syn tg mice

The neurodegenerative pathology (neuronal loss, astrogliosis and demyelination in the corpus callosum) in the MBP-α-syn tg mice is accompanied by behavioral deficits specifically locomotor activity [38] similar to symptoms observed in MSA [3, 39]. In order to investigate the functional effects of the treatment with the LV-NR-R80Q-apoB, mice were examined in the open field test; a behavioral test that examines both anxiety as well as locomotor activity in MSA transgenic mice [38]. At first, total activity after consecutive days was examined; this is a test of habituation to a novel environment and of memory acquisition. The non-tg mice treated with LV-Control or LV-NR-R80Q-apoB rapidly acclimated to the new environment as reflected by the reduced activity over time (Fig. 8a). In contrast the LV-Control-treated MBP-α-syn tg mice did not habituate and activity increased over time, whereas the MBP-α-syn tg treated with LV-NR-R80Q-apoB behaved similar to the non-tg mice (Fig. 8a). These findings were consistent with the retention score analysis, which is the ratio between trials 3 and 4 (Fig. 8b). Next, total spontaneous activity for 10 min was analyzed. As expected, the LV-Control treated MBP-α-syn tg displayed hyper-activity compared to non-tg, however the MBP-α-syn tg mice treated with LV-NR-R80Q-apoB behaved similar to the non-tg controls (Fig. 8c). No differences were noted among the 4 groups in time expended in the center of the cage (Fig. 8d). These results further suggest that gene therapy using a modified, brain-targeted neurosin have beneficial effects on cognition in this tg mouse model of MSA.

## Discussion

We have developed a novel brain penetrating neurosin protein for systemic delivery to the CNS with the potential for treatment of synucleinopathies. In addition to the stabilizing point mutation (R80Q), our fusion protein contained a C-terminal apoB tag that facilitates trafficking of the protein into the CNS via the LDL-R. We showed that the LV-NR-R80Q-apoB reduced the accumulation of α-syn and the propagation of α-syn from oligodendrocytes to astroglia, ameliorated the neurodegenerative pathology including myelination and reversed the behavioral deficits in the MBP-α-syn tg mouse model of MSA. This is the first application of this stabilized neurosin for a therapeutic purpose and represents a novel approach for treatment of a unique synucleinopathy that affects to a greater extent the oligodendrocytes. We chose to test the NR-R80Q-apoB in an MSA model because this construct distributes in multiple brain regions and in MSA there is more diffuse and widespread disease when compared to other synucleinopathies such as PD.

Although neurosin is predominantly expressed in the CNS, many kallikreins exhibit aberrant expression in peripheral tumors such as those of breast and prostate. Interestingly, in both these tumors there is a reduced expression of the kallikreins PSA, kallikrein 6 and NES1 that is associated with cell outgrowth, suggesting that kallikrein expression may have an anti-tumor function in these tissues. In fact, recent investigation of KLK5 has shown that expression in breast tissue regulates several anti-oncogenic miRNAs [40, 41]. In the CNS, neurosin displays remarkably restricted substrate specificity, lacking chymo-, trypsin-, urokinase-, or elastase- specific substrate specificity. In fact, other than α-syn, the primary CNS substrate appears to be PAR-2 where cleavage occurs at S37 to activate the receptor; however, neurosin fails to activate PAR-1 or PAR-4. Activation of PAR-2 by excess neurosin could be potentially harmful to neurons as it elicits elevations in intracellular Ca^+^ levels [42], and elevated intracellular Ca^+^ has been shown to be neurotoxic in cultured hippocampal neurons [43]. However, in this study examination of neuronal cells immunolabeled with NeuN did not show any significant loss of neurons following systemic NR-R80Q-apoB; on the contrary, we found a rescue effect. Further studies should examine off-target effects such as these.

Neurosin protein expression is observed in oligodendrocytes with reduced expression during demyelination [21, 44] and knockout of neurosin reduces MBP protein in the spinal cord of experimental mice indicating that neurosin regulates myelination via MBP [45]. Although the exact role of neurosin activity on myelination or maturation of MBP is not known, we did observe an increase in myelination and MBP following delivery of the recombinant NR-R80Q-apoB to the MBP-α-syn tg mouse. This is consistent with the possibility that neurosin might improve the demyelination phenotype in the mice by modulating MBP. This was observed both by electron microscopy and LFB staining of the corpus callosum. Interestingly, we did not observe increased neurosin protein in the oligodendrocytes; however, we did observe a reduction in the number of α-syn positive oligodendrocytes in the corpus callosum. This is probably due to the lack of LDL-R on these cells preventing the uptake of the neurosin-apoB fusion protein. In addition, we did not observe a change in the expression of the MBP mRNA, suggesting that the change in MBP we observe is at the post-transcriptional level. The increased MBP in the corpus callosum may be due to effects of extracellular neurosin directly on MBP either by stabilizing or by processing the protein. Alternatively, the increased MBP may be due to an overall increase in the overall health of oligodendrocyte cells due to an improvement in the extracellular environment caused by the reduction of extracellular α-syn by neurosin.

**Fig. 9:**
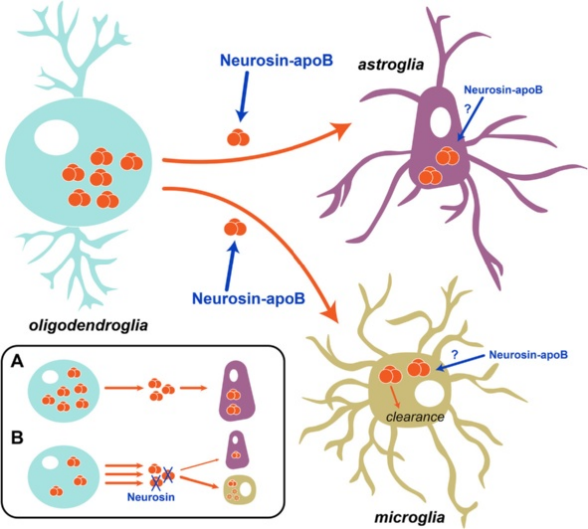
Hypothesis of the effects of treatment with LV-NR-R80Q-apoB in MBP-α-syn tg mice. In the MBP-α-syn tg model of MSA, oligodendrocytes express high levels of α-syn and release it to the extracellular environment, where it can propagate to astroglial cells or be taken up by microglial cells for degradation. The modified, brain-targeted fusion protein NR-R80Q-apoB (neurosin-apoB) is able to access and degrade extracellular α-syn, as well as be internalized by astroglia and microglia, potentially degrading also intracellular α-syn in those cell types. In LV-Control-treated tg mice (**a**), α-syn propagates and accumulates within astroglial cells. However in LV-NR-R80Q-apoB-treated tg animals neurosin degrades extracellular α-syn (**b**), thus stimulating further release of α-syn from oligodendroglial cells (“sink effect”), reducing its propagation and accumulation in astroglia, and inducing its uptake and degradation by microglia

Neurosin is a soluble extracellular protease without a native receptor-binding domain [18]. The addition of the LDL-R binding domain of apolipoprotein B not only targets the recombinant protein for transport across the BBB, but also for uptake by cells expressing the LDL-R. These include neurons, astrocytes and microglial cells and to a much lesser extent oligodendrocytes as evidenced by the LDL-R immunolabeling in this study. We have previously shown similar penetration into the CNS via LDL-R in endothelial cells in the BBB with other proteases such as neprilysin (amyloid β degrading enzyme) [30, 31] and with single chain antibodies targeting α-syn oligomers [29]. For the present study, we showed that delivery of brain-targeted neurosin resulted in decreased α-syn accumulation in oligodendrocytes and astrocytes with a concomitant localization in microglia. Given that in our model α-syn is only expressed in oligodendroglial cells, but is localized in astrocytes, one potential hypothesis for the reduction is that neurosin decreases the transfer of α-syn from oligodendrocytes to astroglia with clearance via microglia. The reduced α-syn transfer could be related to a reduction in extracellular α-syn (“*sink effect*”) (Fig. 9a, b). In support of this possibility we show that microglial cells are able to degrade α-syn in the presence of neurosin. This is consistent with previous studies showing that among other CNS cells, microglial cells are most effective at clearing α-syn [46–48]. Furthermore, the fact that neurosin is also able to penetrate astroglial and microglial cells through interaction of the apoB domain with LDL-R suggest that neurosin might also be degrading intracellular α-syn in those cell types in addition to extracellular α-syn (Fig. 9). Therefore without specific intracellular oligodendrocyte α-syn degradation, we still observed reduced accumulation of α-syn in the brain, reduced astrocyte α-syn uptake and/or accumulation, and improved oligodendrocyte health as observed by increased myelin and MBP expression. A further benefit of decreased extracellular α-syn would be decreased activation of astroctyes [49].

Without specific evidence that neurosin is degrading α-synuclein extracellularly or intracellularly, we cannot definitively rule out other hypotheses and it is clear that further experimental data is needed to better understand how neurosin reduces α-syn in astrocytes. However, neurosin taken up by astrocytes via the LDL-R would be targeted to the lysosome through native LDL-R pathway leading to an acidic environment [50]. Neurosin is known to function best in a neutral environment [51] providing further evidence that little if any α-synuclein degradation would be occurring in astrocyte lysosomes and instead most would be occurring in the extracellular environment.

Taken together these findings suggest that this might be the result of a combination of reduced extracellular α-syn and reduced propagation of α-syn. Interestingly, double labeling studies showed co-localization of α-syn to microglial cells in the MBP-α-syn mice treated with LV-NR-R80Q-apoB, suggesting that in the presence of neurosin, extracellular α-syn was cleared via the microglia. This is consistent with previous studies using anti-α-syn antibodies showing that microglial cells are capable of clearing α-syn [46–48]. A recent study showed that neurosin had reduced degradation of extra-cellular α-syn when in the presence of lipids or fatty acids [52]. The reduction in total α-syn we observed might be due to higher than normal levels of neurosin in the extra-cellular environment leading to proteolysis, increased degradation by microglia or increased intra-cellular proteolysis in astrocytes.

## Conclusion

Considering many synucleinopathies involve the accumulation of α-syn in neurons, astrocytes and oligodendroglial cells and involve the propagation of α-syn from cell-to-cell, the use of NR-R80Q-apoB should be considered a potential therapeutic for other synucleinopathies such as PD and DLB. Systemic delivery of the recombinant protein by intra-peritoneal infusion followed by transport across the BBB for treatment of the whole brain would facilitate a non-invasive and broad-based therapeutic option for numerous α-syn related pathologies.

## Methods

### Construction of lentivirus vectors

The mouse Neurosin cDNA (Open Biosystems) was PCR amplified and cloned into the third generation self-inactivating lentivirus vector [53] with the CMV promoter driving expression producing the vector LV-Neurosin as previously described [25]. The lentivirus vector expressing the human wild-type α-syn has been previously described. The R80Q neurosin mutation was generated as described [26] using the QuikChange Lightning Site-Directed Mutagenesis Kit (Agilent Technologies). Addition of the 38 amino acid apoB LDL-R binding domain to the c-terminus of both wild-type neurosin and the R80Q neurosin was performed by site directed mutagenesis addition of a unique AgeI restriction site in frame followed by cloning of the oligonucleotide coding for the apoB peptide [29, 30, 32]. Lentiviruses expressing the neurosin (LV-wt-NR), neurosin-apoB (LV-wt-NR-apoB), neurosin R80Q (LV-NR-R80Q), neurosin R80Q-apoB (LV-NR-R80Q-apoB, α-synuclein (LV-α-syn) or empty vector (LV-Control) were prepared by transient transfection in 293T cells [53].

### Establishment of *in vitro* cultures

For these experiments we used the rat neuroblastoma cell line B103. This model was selected because overexpression of α-syn in these cells results in mitochondrial alterations, reduced cell viability, defective neurite outgrowth and abnormal accumulation of oligomeric α-syn [54]. For all experiments, cells were infected with LVs at a multiplicity of infection (MOI) of 20.

The B103 cells were grown as described above and plated onto poly L-lysine coated glass coverslips at a density of 5 × 10^4^ cells. Five hours after plating, cells were infected with the LV-Neurosin vectors and incubated for 48 h. Control experiments were performed with cells infected with LV-Control. For co-culture analysis, 5 × 10^4^ B103 cells were plated onto poly L-lysine coated glass coverslips and infected with LV-Neurosin vectors or onto 12 well cell culture inserts containing a 0.4 μm PET membrane (Fisher Scientific) and infected with LV-α-syn vectors. Cultures were incubated separately for 6 h to allow cells to attach and then co-cultured until analysis.

BV2 microglia cells were grown as previously described [46] and plated on poly L-lysine coated glass coverslips at a density of 5 × 10^4^ cells for 24 h. Cultured supernatent from LV-NR-R80Q-apoB or LV-Control infected B103 cells was collected and passed through a 0.22 μm filter to remove any cells. The supernatent was then incubated with 500nM of previously oligomerized α-syn [25] for 32 h at 37 °C and then added to BV2 cells for 1 h at 37 °C, 5 % CO_2_. At the conclusion of all experiments, cells were washed twice with ice-cold PBS followed by fixation with cold 4 % PFA.

### Treatment of animals

Mice expressing human α-syn under the control of the Myelin Basic Protein (MBP) promoter (MBP-α-syn tg) were generated as previously described [27]. In this study we used the MBP1 line, as animals express an intermediate level of α-syn compared to the other lines and they are more viable and less aggressive. MBP-α-syn tg mice develop progressive accumulation of α-syn inclusions in oligodendrocytes along the axonal tracts in the brainstem, basal ganglia, cerebellum, corpus callosum, and neocortex, leading to neurodegeneration in the neocortex and to loss of dopaminergic fibers in the basal ganglia. Non-tg and MBP-α-syn tg mice received a single intra-peritoneal injection of LV-Control (1.0 × 10^9^ TDU/mouse) or LV-NR-R80Q-apoB (1.0 × 10^9^ TDU/mouse) (*n* = 10/group) in a volume of 300 μl. We, and others have previously shown that this method of vector delivery results in lentiviral transduction of the liver (sinusoidal cells and to a lesser extent hepatocytes) and the spleen (splenocytes) where the secreted protein is expressed and transported by the blood to the blood–brain barrier [32, 55]. Treatment lasted 3 months with all mice between 9 and 10 months of age by the end of the study. All experiments were carried out in accordance with the guidelines laid down by the NIH regarding the care and use of animals for experimental procedures and approved by the Institutional Animal Care and Use Committee at the University of California at San Diego (UCSD) under protocol #S02221.

### Behavioral analysis

Context-dependent learning in an open field was analyzed using a Kinder SmartFrame Cage Rack Station activity monitor system (Kinder Scientific, Poway, CA), in 3-dimensional space using a 7 × 15 beam configuration. Data collection began when an animal was placed in the test chamber. Animals were evaluated for 10 min for three consecutive days, given a two-day dishabituation period, followed by a fourth trial [56].

### Real-time PCR analysis

Total RNA was extracted from the mouse anterior hemibrain using a Qiagen RNeasy kit and following the instructions of the manufacturer. RNA concentration was determined and 0.5 μg of RNA per sample were used for reverse transcription to cDNA using a High capacity cDNA reverse transcription kit (Applied Biosystems). cDNA solutions were diluted 1:10 in ultrapure water and 4 μl of this dilution were used per reaction. Real time PCR (qPCR) was performed using Fast SYBR Master Mix and primers for human α-syn, mouse MBP, TNFα, IL-1ß, IL-6, IL-10 and ß-actin as internal control [35, 57]. qPCR reactions were run in a StepOnePlus Real-Time PCR system (Applied Biosystems) and ΔΔCt calculations were made using StepOne software (Applied Biosystems).

### Immunoblot analysis

Hemibrains were homogenized and divided into cytosolic and membrane fractions as previously described [54, 58]. For immunoblot analysis, 20 μg of total protein per lane was loaded on 4–12 % Bis-Tris SDS-PAGE gels and blotted onto polyvinylidene fluoride membranes. Membranes were probed with antibodies against full-length human α-syn (SYN211, Life Technologies), human neurosin (R&D Systems) or the epitope tag V5 (Life Technologies). Incubation with primary antibody was followed by species-appropriate incubation with secondary antibody tagged with horseradish peroxidase (Santa Cruz Biotechnology) and visualization with enhanced chemiluminescence.

Heparinized blood (plasma) (diluted 1:10 in PBS) and CSF (undiluted) samples were loaded on 4-12 % Bis-Tris SDS-PAGE gels and blotted onto polyvinylidene fluoride membranes and probed with antibodies against human neurosin (R&D Systems) as described above. Analysis of all immunoblot was performed with a Versadoc XL imaging apparatus (BioRad) using β-actin (Sigma) levels as a loading control.

### Immunohistochemistry and electron microscopy

At the end of the experiment, CSF and blood were collected and animals were transcardially perfused with physiological saline and brains were collected. Brains were divided sagittally into right and left hemibrains. The left hemibrain was fixed in 4 % paraformaldehyde in phosphate buffered saline and serially sectioned in the vibratome at 40 μm and stored at −20 °C in cryoprotective medium. The right hemibrain was snap-frozen and stored at −80 °C for subsequent protein extraction. Sections were immunostained with antibodies against α-syn (Chemicon), NeuN (neuronal marker, Millipore), GFAP (astroglial marker, Millipore), Iba-1 (microglia, Wako), Olig-2 (oligodendrocytes, Cell Signaling) and p25 (oligodendrocytes, IBL) and imaged with an Olympus BX54 bright field digital microscope or a laser scanning confocal microscope. Digital images were analyzed with the ImageQuant 1.43 program (NIH) to determine numbers of α-syn aggregates, neurons, astrogliosis and microgliosis [28, 46]. A minimum of 100 cells with 4 fields were counted per animals and expressed as the average number of positive cells per field (230 μm × 184 μm). Stereological analysis of NeuN, Iba-1 and α-syn immunoreactivity was conducted by the dissector method using the Stereo-Investigator System (MBF Bioscience), and the results were averaged and expressed as cell counts per 0.1 mm^3^. Additional sections were stained with luxol fast blue (LFB, Acros) in order to visualize the myelin layers and imaged on the Olympus BX54 bright field digital microscope [59]. Optical density measurements were obtained using the ImageQuant software, and quantification performed by correcting against background signal levels.

For electron microscopy, vibratome sections were postfixed in 1 % glutaraldehyde, treated with osmium tetraoxide, embedded in epon araldite and sectioned with the ultramicrotome (Leica). Grids were analyzed with a Zeiss OM 10 electron microscope as previously described [54]. Cells were randomly acquired from 3 grids, and electron micrographs were obtained at a magnification of 5,000X and 25,000X.

### Double immunolabeling and confocal microscopy

To determine the co-localization between α-syn or neurosin and cellular markers, double-labeling experiments were performed as previously described [25]. Vibratome sections were immunolabeled with antibodies against S100 (astroglia), Iba1 (microglia), Olig-2 (oligodendrocytes), p25 (oligodendrocytes) or NeuN (neurons), and the immunoreactive structures were detected with FITC-tagged secondary antibody (Vector Laboratories, 1:75) and the V5 tagged neurosin with the Tyramide Signal Amplification™-Direct system (NEN Life Sciences), while α-syn was detected with an antibody specific for human α-syn (SYN211, Sigma) and a FITC-tagged secondary antibody (Vector Laboratories, 1:75). Cell nuclei were stained using ProLong® Gold Antifade with DAPI (4',6-diamidino-2-phenylindole) (Molecular Probes). Sections were imaged with a Zeiss 63X objective on an Axiovert 35 microscope (Zeiss) with an attached MRC1024 laser scanning confocal microscope (BioRad) with an optical image of 1 μm thick with fluorescent signals in co-registry [60].

### Statistical analysis

Values are expressed as average ± standard error of the mean (SEM). To determine the statistical significance we used one-way analysis of variance with post-hoc Dunnett’s test when comparing to the control condition. Additional comparisons were done using Tukey-Kramer or Fisher post hoc tests. The differences were considered to be significant if p values were less than 0.05.

## Additional files

Additional file 1: Figure S1 Analysis of LDL-R expression in the brain of MBP-α-syn tg mice. Vibratome brain sections were analyzed by double immunofluorescence and confocal microscopy for analysis of the co-localization between LDL-R (red) and cellular markers (green). Dotted box to the left depicts the image field zoomed represented under detail. (A) Laser scanning confocal microscopy of sections from MBP-α-syn tg immunostained with LDL-R (red) and antibodies against NeuN (neurons), p25 (oligodendrocytes), GFAP (astrocytes) and Iba-1 (microglia) (red). (B) Computer aided image analysis for the % of cells displaying co-localization of LDL-R and cellular markers. *n* = 10 mice per group. Scale bar = 10 μm. (TIFF 3556 kb) Additional file 2: Figure S2 Neurosin co-localizes with α-syn in the striatum. Laser scanning confocal microscopy analysis of sections double labeled with antibodies against V5 tagged neurosin (red) and α-syn (green) in the striatum of non-tg and MBP-α-syn tg mice injected with LV-Control or LV-NR-R80Q-apoB. Dotted box to the left depicts the image field zoomed represented under detail. Scale bar = 10 μm. *n* = 10 mice per group 9–10 m/o at the end of the treatment. (TIFF 1909 kb) Additional file 3: Figure S3 Real time PCR analysis of MBP and α-syn expression in MBP-α-syn tg mice. Real-time PCR analysis of gene expression in non-tg and MBP-α-syn tg mouse brain tissue after treatment with LV-Control or LV-NR-R80Q-apoB. Total RNA was extracted and used for real-time PCR analysis using either primers specific for (A) MBP or (B) human α-syn. Gene expression signal was normalized to β-actin signal. *n* = 4 mice per group. (TIFF 371 kb) Additional file 4: Figure S4 Levels of α-syn immunoreactivity in individual oligodendroglial cells in the brains of MBP-α-syn tg that received LV-NR-R80Q-apoB. Vibratome brain sections were analyzed by double immunofluorescence and confocal microscopy for analysis of the levels of human α-syn (red) in oligodendroglial cells labeled with an antibody against p25 (green). Dotted box to the left depicts the image field zoomed represente d under detail. (A) Laser scanning confocal microscopy of sections from non-tg and MBP-α-syn tg immunostained with human α-syn (red) and p25 (green). (B, C) Computer aided image analysis of p25 positive oligodendrocytes expressed as numbers per 10^3^ in the striatum and of the levels of α-syn in individual p25 positive cells expressed as pixel intensity. *n* = 10 mice per group. Scale bar = 30 μm. (TIFF 2838 kb) Additional file 5: Figure S5 Microglia endocytose extracellular α-syn digested by neurosin. BV2 microglia cells were analyzed by double immunofluorescence and confocal microscopy for Iba1 (green) and α-syn (red). BV2 cells were incubated with oligomeric α-syn with or without pre-incubation with cultured supernatent from LV-NR-R80Q-apoB infected B103 neuronal cells. Dotted box to the left depicts the image field zoomed represented under detail. Scale bar = 20 μm. (TIFF 8074 kb) Additional file 6: Figure S6 NR-R80Q-apoB does not significantly affect expression levels of CNS cytokines. Real-time PCR analysis of gene expression in non-tg and MBP-α-syn tg mouse brain tissue after treatment with LV-Control or LV-NR-R80Q-apoB. Total RNA was extracted and used for real-time PCR analysis using either primers specific for (A) TNFα, (B) IL-1ß, (C) IL-6 or (D) IL-10. Gene expression signal was normalized to β-actin signal. *n* = 4 mice per group. (TIFF 4474 kb)
