# Supplementary figures and images for: A brain-targeted, modified neurosin (kallikrein-6) reduces α-synuclein accumulation in a mouse model of multiple system atrophy
Source: Mol Neurodegener. 2015 Sep 23;10:48. doi: 10.1186/s13024-015-0043-6 (PMC4580347; doi:10.1186/s13024-015-0043-6)

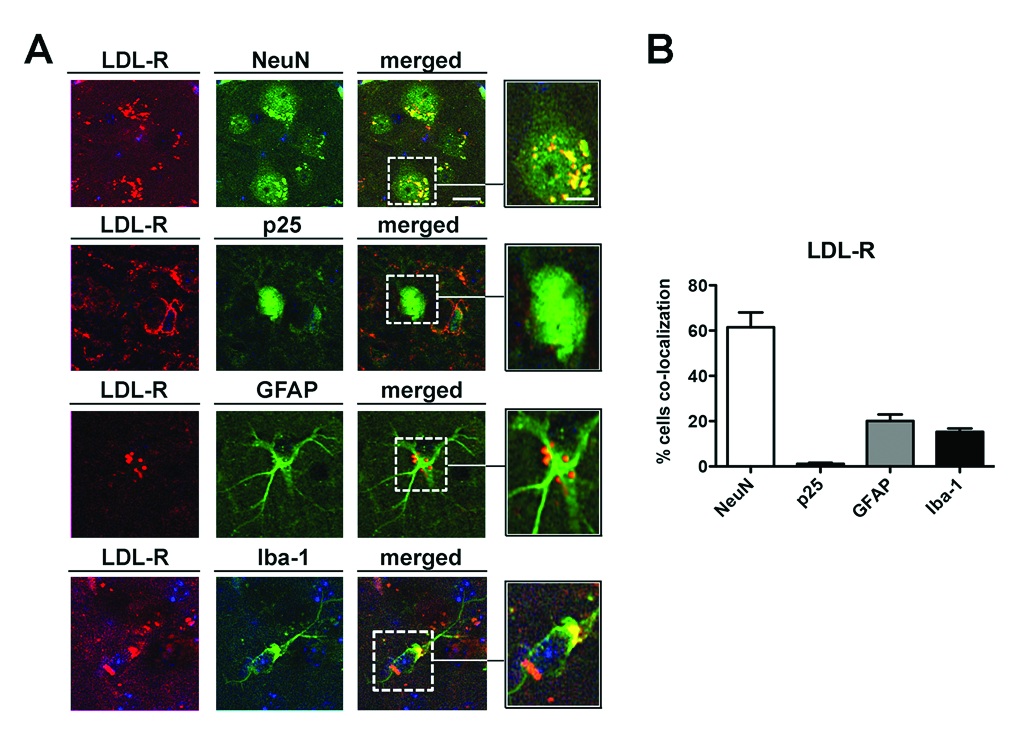

Supplement: Additional file 1: Figure S1 — Analysis of LDL-R expression in the brain of MBP-α-syn tg mice. Vibratome brain sections were analyzed by double immunofluorescence and confocal microscopy for analysis of the co-localization between LDL-R (red) and cellular markers (green). Dotted box to the left depicts the image field zoomed represented under detail. (A) Laser scanning confocal microscopy of sections from MBP-α-syn tg immunostained with LDL-R (red) and antibodies against NeuN (neurons), p25 (oligodendrocytes), GFAP (astrocytes) and Iba-1 (microglia) (red). (B) Computer aided image analysis for the % of cells displaying co-localization of LDL-R and cellular markers. n = 10 mice per group. Scale bar = 10 μm. (TIFF 3556 kb) [file 13024_2015_43_MOESM1_ESM.tif]

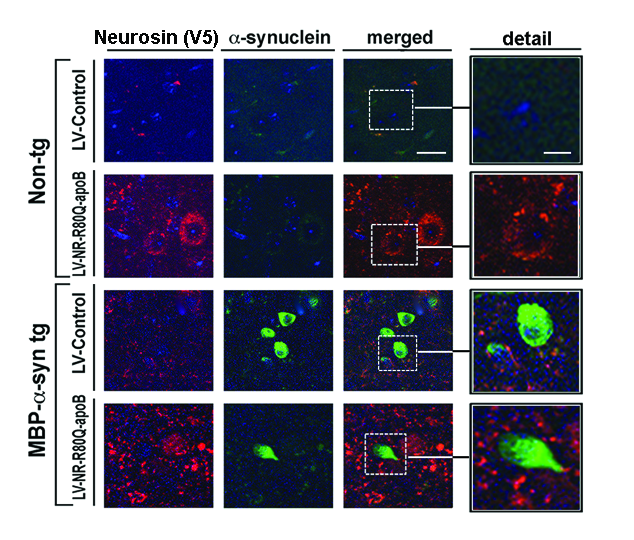

Supplement: Additional file 2: Figure S2 — Neurosin co-localizes with α-syn in the striatum. Laser scanning confocal microscopy analysis of sections double labeled with antibodies against V5 tagged neurosin (red) and α-syn (green) in the striatum of non-tg and MBP-α-syn tg mice injected with LV-Control or LV-NR-R80Q-apoB. Dotted box to the left depicts the image field zoomed represented under detail. Scale bar = 10 μm. n = 10 mice per group 9–10 m/o at the end of the treatment. (TIFF 1909 kb) [file 13024_2015_43_MOESM2_ESM.tif]

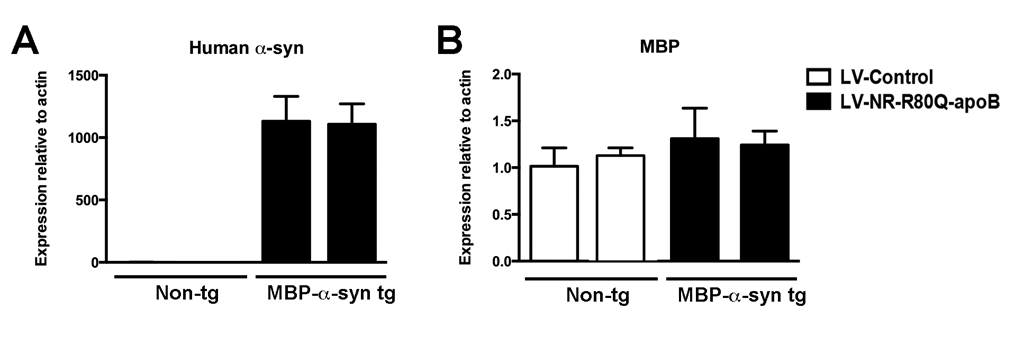

Supplement: Additional file 3: Figure S3 — Real time PCR analysis of MBP and α-syn expression in MBP-α-syn tg mice. Real-time PCR analysis of gene expression in non-tg and MBP-α-syn tg mouse brain tissue after treatment with LV-Control or LV-NR-R80Q-apoB. Total RNA was extracted and used for real-time PCR analysis using either primers specific for (A) MBP or (B) human α-syn. Gene expression signal was normalized to β-actin signal. n = 4 mice per group. (TIFF 371 kb) [file 13024_2015_43_MOESM3_ESM.tif]

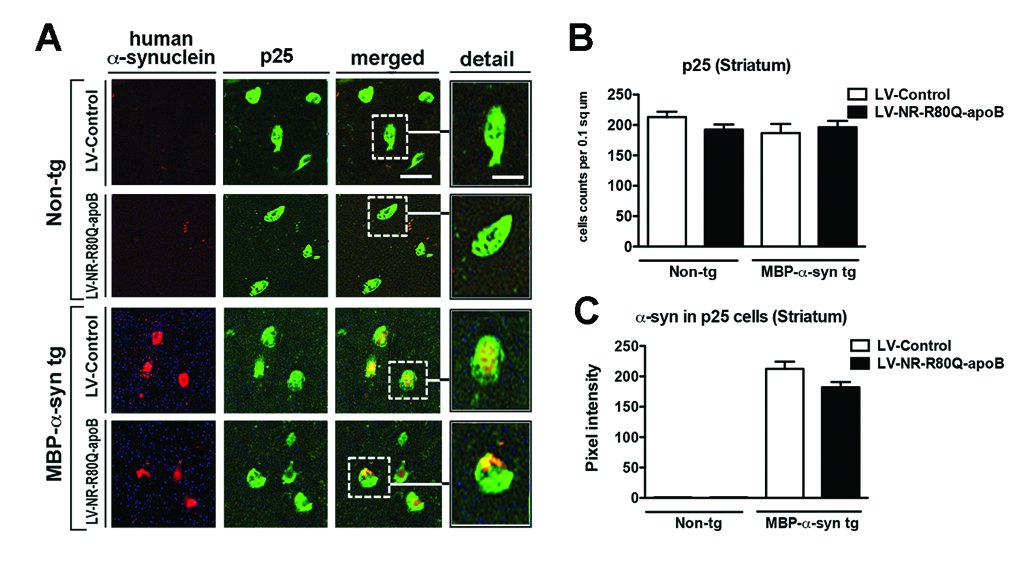

Supplement: Additional file 4: Figure S4 — Levels of α-syn immunoreactivity in individual oligodendroglial cells in the brains of MBP-α-syn tg that received LV-NR-R80Q-apoB. Vibratome brain sections were analyzed by double immunofluorescence and confocal microscopy for analysis of the levels of human α-syn (red) in oligodendroglial cells labeled with an antibody against p25 (green). Dotted box to the left depicts the image field zoomed represente d under detail. (A) Laser scanning confocal microscopy of sections from non-tg and MBP-α-syn tg immunostained with human α-syn (red) and p25 (green). (B, C) Computer aided image analysis of p25 positive oligodendrocytes expressed as numbers per 103 in the striatum and of the levels of α-syn in individual p25 positive cells expressed as pixel intensity. n = 10 mice per group. Scale bar = 30 μm. (TIFF 2838 kb) [file 13024_2015_43_MOESM4_ESM.tif]

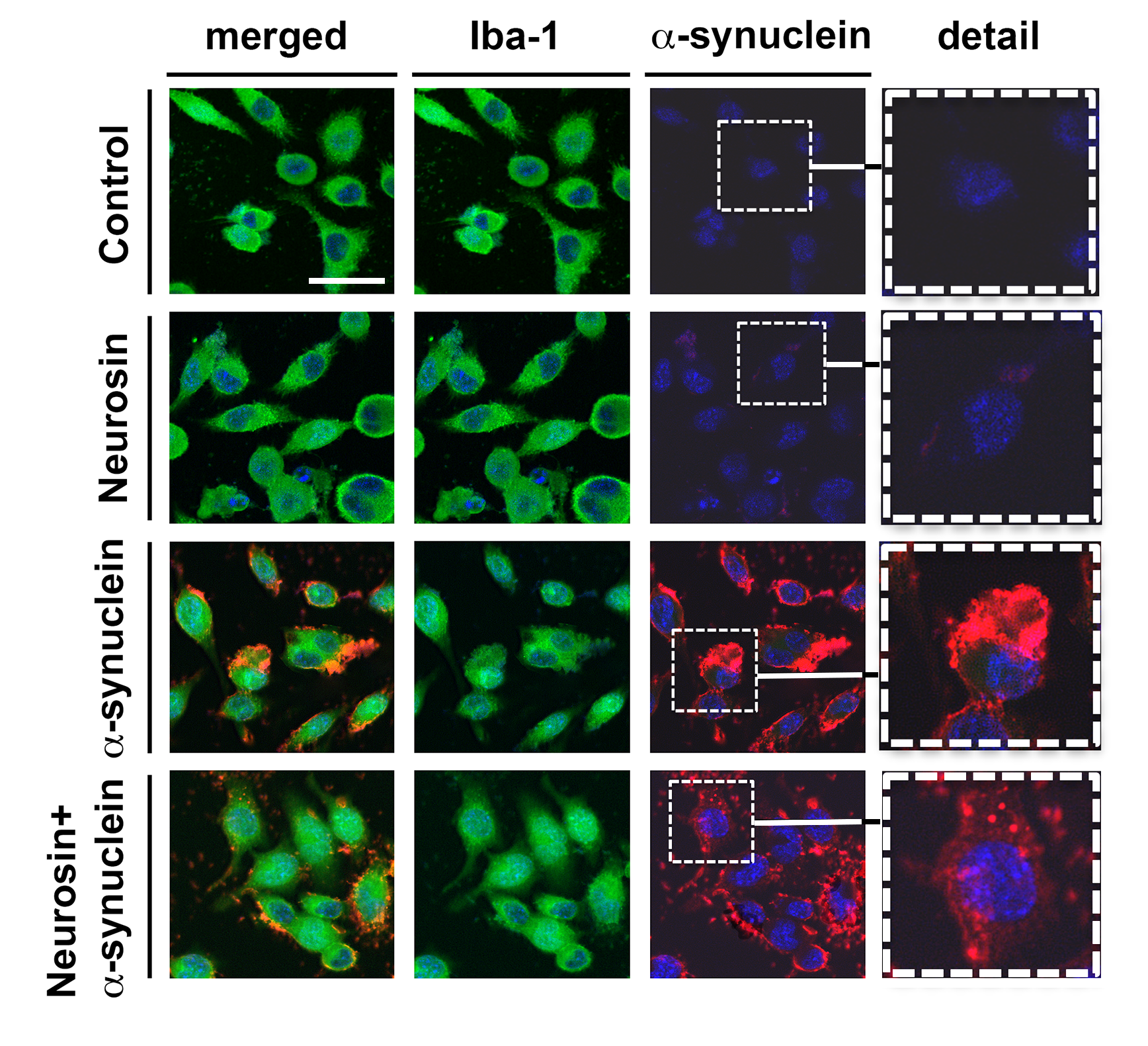

Supplement: Additional file 5: Figure S5 — Microglia endocytose extracellular α-syn digested by neurosin. BV2 microglia cells were analyzed by double immunofluorescence and confocal microscopy for Iba1 (green) and α-syn (red). BV2 cells were incubated with oligomeric α-syn with or without pre-incubation with cultured supernatent from LV-NR-R80Q-apoB infected B103 neuronal cells. Dotted box to the left depicts the image field zoomed represented under detail. Scale bar = 20 μm. (TIFF 8074 kb) [file 13024_2015_43_MOESM5_ESM.tif]

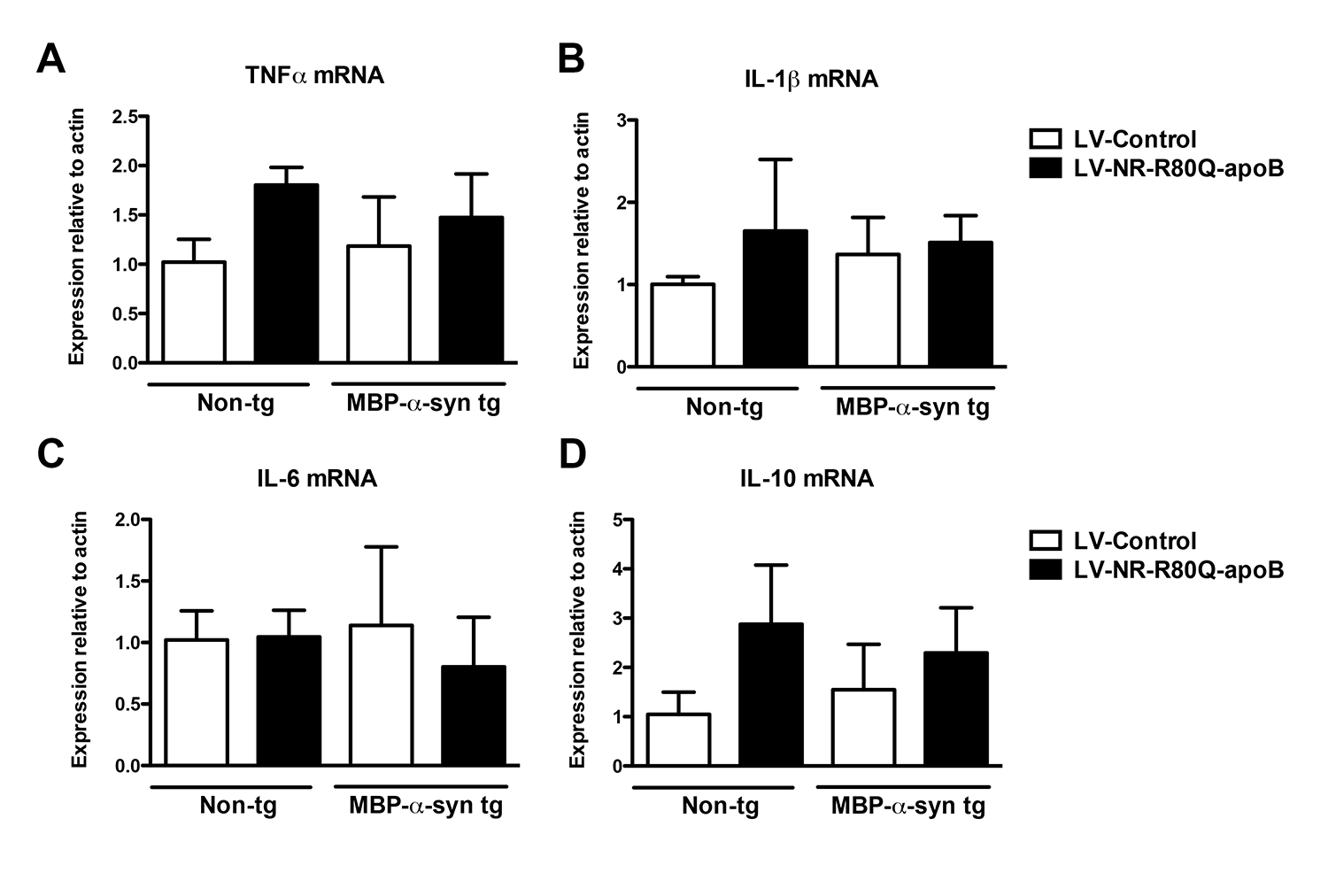

Supplement: Additional file 6: Figure S6 — NR-R80Q-apoB does not significantly affect expression levels of CNS cytokines. Real-time PCR analysis of gene expression in non-tg and MBP-α-syn tg mouse brain tissue after treatment with LV-Control or LV-NR-R80Q-apoB. Total RNA was extracted and used for real-time PCR analysis using either primers specific for (A) TNFα, (B) IL-1ß, (C) IL-6 or (D) IL-10. Gene expression signal was normalized to β-actin signal. n = 4 mice per group. (TIFF 4474 kb) [file 13024_2015_43_MOESM6_ESM.tif]
